# Spatial distribution and associated factors of zero-dose immunization among 12–23 month-old children in Ethiopia: spatial and survey regression analysis

**DOI:** 10.1186/s12887-025-05900-5

**Published:** 2025-07-14

**Authors:** Kindie Fentahun Muchie, Abebaw Gedef Azene, Kassawmar Angaw Bogale, Desale Bihonegn Asmamaw, Wubshet Debebe Negash, Tadele Biresaw Belachew, Bethelihem Tigabu Tarekegn, Bewuketu Terefe, Getasew Mulat Bantie, Habitu Birhan Eshetu, Gizachew Tadesse Wassie

**Affiliations:** 1 Department of Epidemiology and Biostatistics, School of Public Health, College of Medicine and Health Sciences, Bahir Dar University, Bahir Dar, Ethiopia; 2 Department of Infectious Disease and Tropical Medicine, Heidelberg University Hospital, Heidelberg, Germany; 3 Division of Epidemiology and Biostatistics, School of Public Health, Faculty of Health Sciences, University of Witwatersrand, Johannesburg, Republic of South Africa; 4 Department of Reproductive Health, Institute of Public Health, College of Medicine and Health Sciences, University of Gondar, Gondar, Ethiopia; 5 Monash Centre for Health Research and Implementation, Faculty of Medicine, Nursing and Health Sciences, Monash University, Melbourne, Australia; 6 Department of Health Systems and Policy, Institute of Public Health, College of Medicine and Health Sciences, University of Gondar, Gondar, Ethiopia; 7 National Centre for Epidemiology and Population Health, The Australian National University, Canberra, Australia; 8 Department of Pediatrics and Child Health Nursing, School of Nursing, College of Medicine and Health Sciences, University of Gondar, Gondar, Ethiopia; 9 Department of Community Health Nursing, School of Nursing, College of Medicine and Health Sciences, University of Gondar, Gondar, Ethiopia; 10 Amhara National Regional State Public Health Institute, Bahir Dar, Ethiopia; 11 Department of Health Promotion and Health Behavior, Institute of Public Health, College of Medicine and Health Sciences, University of Gondar, Gondar, Ethiopia

**Keywords:** Zero-dose, Immunization, Prevalence, Spatial distribution, Associated factors, Survey regression, Ethiopia

## Abstract

**Background:**

Immunization is one of the public health interventions, saving millions of lives. Despite this, many children, especially those living in low- and middle-income countries, continue to miss out on lifesaving vaccines, the worst of which is zero-dosage. It is crucial to identify individuals and obtain timely, reliable information on their geographic distribution and related attributes to support spatially tailored strategies and interventions. Undoubtedly, assessing zero-dose prevalence is an input to achieve the WHO’s 2030 agenda, which attempts to reduce the number of zero-dose children. Hence, this study aimed to determine the prevalence, spatial distribution and associated factors of zero-dose immunization among children in Ethiopia.

**Methods:**

A secondary analysis of the Ethiopian mini demographic and health survey 2019 data was conducted. Our analysis focused on zero-dose immunization among children aged 12–23 months. Geographic variations in zero-dose prevalence were assessed using spatial analysis techniques, including Moran’s I statistic and inverse distance weighted interpolation. Bivariable and multivariable survey logistic regression models were used to identify factors associated with zero-dose immunization.

**Results:**

A total of 1008 children aged 12 to 23 months old were retained for the final analysis. The overall weighted prevalence of zero-dose immunization status at national level in Ethiopia was 23.7% [95% CI: 18.7–28.5]. Hot spots of zero-dose immunization were observed in southwest and northeast part of Ethiopia whereas cold spots of zero-dose immunization were observed in the central and northern parts of the country. Rural resident children [AOR = 2.55; 95%CI: 1.05, 6.22], female children (AOR = 1.78; 95%CI: 1.09, 2.91), children not delivered at the health institution (AOR = 4.12; 95% CI: 2.39, 7.08), and children from a mother did not completed four or more ANC visits (AOR = 2.55; 95% CI: 1.37, 4.75) were more likely to be zero-dose as compared to their counter parts.

**Conclusion:**

There is high prevalence of children being zero-dose among 12–23 months old children in Ethiopia. Interventions tailored on geographic areas, residence, sex of the child, four or more ANC visits and institutional delivery could help to reduce zero-dose children in Ethiopia.

## Background

Immunization is defined as the act of injecting a vaccine into a person’s body to build protection against a specific disease [1]. Besides, children who have not had at least one standard vaccination are considered “zero-dose” according to the World Health Organization (WHO). For operational purposes, zero-dose children are defined as those who miss the first dose of the diphtheria, tetanus, and pertussis containing vaccine (DTP1) [2]. The GAVI’s definition helps target the most vulnerable groups, often excluded from basic vaccines, while the WHO definition covers a broader range of missed vaccines. The former definition aligns more closely with the goals of vaccination campaigns in resource-limited settings like Ethiopia, enabling more effective efforts to reduce childhood morbidity and mortality.

Immunization is among the most cost-effective ways to improve health and well-being throughout the life course [3]. It plays a role as an entry point into primary health care and keeps the disease from spreading when people interact with others and their environment [3]. With all these points, lack of access to vaccines leaves children at risk of death, disability, and illness from preventable diseases [4]. Despite intensified efforts worldwide to lower zero-dose prevalence, many children still do not receive life saving vaccines [5]. Accordingly, one in five children globally did not have access to essential immunizations in 2023 [3, 4]. Fourteen and a half (14.5) million infants were zero-dose children, and an additional 6.5 million are partially vaccinated [4]. Of these, 21 million children, 60% live in just 10 countries of low- and middle-income countries (LMICs) including Ethiopia, and almost all zero-dose children live in LMICs [4].

Routine data sources can often be incomplete and unreliable in many LMICs. Sub-national estimates of the indicators are produced regularly using nationally representative household surveys such as the demographic and health surveys which are typically conducted every five years. The latest survey, the Ethiopian mini-Demographic and Health Survey (EMDHS) 2019 reported that nearly 44% of children aged 12–23 months have received all basic vaccinations at some time [6]. The percentage of children who received all basic vaccinations has increased by 5% since 2016 (from 39 to 44%) [6, 7] with rates varying greatly across geographical regions [7]. Though there has been observed decline in proportion of children with no vaccinations from 24% in 2005 to 19% in 2019 it has increased from 16% in 2016 to 19% in 2019 [6].

Zero-dose children oftentimes reside in under-privileged or marginalized neighbourhoods that are characterized by conflict, overcrowding, inadequate sanitation, and limited access to basic health services [8–11]. These conditions, along with other health-related, socioeconomic, demographic, and gender-related factors, contribute to significant within- country disparities in the distribution of zero-dose children [9]. Therefore, it is imperative to identify these at-risk groups to support country-tailored strategies and interventions using timely and reliable knowledge about their geographic distribution and other features. Undeniably, evaluating zero-dose prevalence will help as an input to achieve the WHO’s immunization agenda 2030 target of a 50% reduction in zero-dose children by 2030 [8, 12]. This effort aligns with the global promises to “leave no one behind” and supports the sustainable development goals and Gavi, the Vaccine Alliance’s 2021–2025 Strategy [8, 12].

Health indicators such as immunization coverage are regularly monitored within countries to inform policy and decision-making and evaluate progress towards key development goals. Since the launch of the sustainable development goals [12] in 2015, there has been an increasing recognition of geographical precision in the estimation of the indicators. Spatially detailed distribution and determining the associated factors of the zero-dose immunization taking survey design into account are key to understanding the inequities that exist within the country, which are often masked by country level and regional estimates.

Understanding the spatial distribution of zero-dose children in LMICs is critical for targeting interventions in areas with the greatest need [11, 13, 14]. These children often reside in “immunization deserts,” including remote rural areas, urban slums, and conflict zones, where healthcare access is limited or disrupted [2]. Spatial distribution analyses helps to identify such high-risk regions, enabling health programs to prioritize resource allocation and implement targeted solutions, such as deploying mobile clinics or strengthening local healthcare infrastructure [13].

A comprehensive understanding of factors such as socio-economic, cultural, and other related factors combined with spatial distribution could further amplify the effectiveness of these strategies to overcome cultural and systematic barriers to immunization uptake. Combining geospatial insights with these associated factors can reveal areas where intersecting inequities exacerbate zero-dose prevalence, guiding tailored interventions [13]. For instance, spatial mapping integrated with socio-economic indicators is helpful for designing culturally sensitive education campaigns and addressing infrastructure gaps, resulting in improved vaccination coverage [11]. A combined spatial and factor-based approach is essential for reducing the prevalence of zero-dose children and achieving equitable immunization goals in LMICs.

Several studies have examined zero-dose immunization among children in Ethiopia [15–17] with notable gaps that our study aims to address. Two of these studies [15, 17] considered children aged 12–35 months from vulnerable populations in diverse and hard to reach areas of the country. Furthermore, a study [17] considered only the spatial distribution of zero-dose immunization, while the other [15] focused associated factors only. The third study [16] addresses some of the limitations of the previous studies by including children representing the entire population and considering both spatial distribution along with associated factors. However, the broader age range included in this study may limit its ability to specifically identify children who missed their initial DTP doses.

In addition, including children older than 23 months might introduce potential recall bias and variability from delayed or booster dose vaccinations, which could obscure the primary objective of assessing zero-dose status. Our study uniquely fills these gaps by focusing specifically on children aged 12–23 months, allowing for a more accurate assessment of zero-dose children and strengthening the relevance of findings for targeted interventions.

So far, no study in Ethiopia has examined the spatial distribution of zero-dose immunization and its associated factors simultaneously at the national level while considering sampling weights and design, particularly with a focus on the appropriate age group for children. In light of this, our study aimed to analyze data from children aged 12–23 months by incorporating sampling weights and design to provide valuable insights into the spatial distribution of zero-dose immunization and its contributing factors, which can help inform effective strategies to improve immunization coverage in Ethiopia.

## Methods

### Study setting and design

This study is further analysis based on the Ethiopian Mini Demographic and Health Survey (EMDHS) data, which was collected in March 21 - June 28, 2019.

The 2019 EMDHS is the second mini DHS and the fifth DHS, which is a cross-sectional survey conducted as part of the global DHS Program, formerly known as MEASURE DHS [6]. The DHS program provides technical assistance, advancing global understanding of health and population trends in developing countries [18]. Accordingly, the 2019 EMDHS was implemented by the Ethiopian Public Health Institute, in partnership with the Central Statistical Agency and the Federal Ministry of Health, under the overall guidance of the technical working group including the DHS program [6].

There are geographic disparities and transportation challenges in Ethiopia that could significantly limit healthcare access, especially in sparsely served areas. The median walking time to the nearest health facility is 33 min in accessible areas, but rural regions often face travel times exceeding 2 hours [19]. The Ethiopian health care system has a three-tier system [20]. The first is primary care, which comprises health posts, health centers, and primary hospitals. The next is secondary care, which is composed of general hospitals. The last is tertiary care, which is composed of specialized hospitals. Immunization services are delivered at all levels of health care system tiers in the country.

### Data sources and procedures

The analysis of zero-dose immunization in the current study used the most recent available survey data, the EMDHS 2019. The 2019 EMDHS employed a stratified two-stage cluster sampling technique, selecting 149,093 enumeration areas (EAs) from the 2019 Ethiopia Population and Housing Census, with each enumeration area averaging 131 households [6]. A random sample of households was selected following the listing and mapping of all conventional households within the chosen EAs. Ultimately, a nationally representative sample of 9,150 households were surveyed, identifying 9,012 eligible women for interviews, with all women aged 15–49 years in the selected households considered eligible.

Data on immunization coverage were collected through mother’s verbal reports, vaccination cards, and health facility records [6]. During individual interviews, mothers provided information on vaccinations received by their children born in the three years preceding the survey. For this study, the focus was on zero-dose immunization status for children aged 12–23 months.

The EMDHS 2019 data for the current study were extracted from the DHS Program database(http://dhsprogram.com) after we have received a grant of permission. The survey’s Global Positioning System (GPS) coordinates for the EAs were also obtained and processed with authorization from the DHS Program. To overlay immunization prevalence with Ethiopia’s administrative classifications, freely available second-level administrative boundaries (zones) were downloaded from the Humanitarian Data Exchange website (https://data.humdata.org).

The immunization data relevant for estimating zero-dose immunization prevalence were retrieved from the children’s survey dataset and the GPS points of the EAs of the 2019 EMDHS. All the variables used in this analysis were then retrieved from the children’s data sets of the survey data.

### Study population

The target population for this study comprised of all 12–23-month-old children in Ethiopia. The age range of 12–23 months is widely used for assessing zero-dose immunization because it represents a critical period when children should have completed the primary DTP vaccination series, as recommended by the WHO. Selecting children younger than 12 months might not capture the full impact of immunization gaps, as they may not yet have reached the critical vaccination milestones. Excluding children older than 23 months avoids potential recall bias and variability from delayed or catch-up vaccinations, which could obscure the primary objective of assessing zero-dose status. This age group has been used in numerous similar studies [21–23]. This age group is a standard target for immunization surveys, including those conducted by UNICEF and WHO, as it aligns with efforts to measure immunization coverage and identify gaps in vaccine delivery systems. This focus ensures comparability across global immunization surveys while prioritizing the most vulnerable population for targeted interventions to reduce early childhood morbidity and mortality. Moreover, this age range is standard in vaccination assessments, enabling precise identification of early immunization gaps critical for programmatic efforts.

### Variables of the study

The outcome of interest was zero-dose immunization status (1 = Yes, not received the DTP1 where as 0 = otherwise) among children aged 12–23 months. The potential explanatory factors considered were household factors, mother’s/caregiver’s socio-demographic factors and child socio-demographic factors.

The candidate explanatory variables considered in this study were selected based on the availability of the variable in the survey data and review of related literature. Accordingly, three groups of variables namely household factors, mother’s/caregiver’s factors, and child factors. Accordingly, wealth index, type of toilet facility, type of drinking water source, distance to water source, region, and residence were the household factors where as educational level, marital status, age, religion, antenatal care service utilization, and institutional delivery were included under mother’s/caregiver’s factors while sex of the child was the only child socio-demographic factors.

The variable type of source of drinking of water was categorized into two groups (improved vs. unimproved). The improved includes piped water, public tap/stand pipe, tube well or bore hole, protected dug well, protected spring, rain water, and bottled water whereas the unimproved type includes unprotected dug well, unprotected spring, tanker truck/cart with small tank, and surface water. Furthermore, the time to water source in minutes was grouped into four categories (on premise, 0 to 30 min, 31–60 min and more than or equal to 60 min).

The variable type of toilet facility was grouped into three categories: improved, unimproved, and open field. Accordingly, any non-shared flush or pour flush toilets to a piped sewer system, septic tank, pit latrine, or unknown destination; ventilated improved pit (VIP) latrines; pit latrines with slabs; and composting toilets were considered improved. On the other hand, any toilet type shared by two or more households, including flush/pour flush not to a sewer/septic tank/pit latrine, pit latrines without slabs/open pits, buckets, hanging toilets/hanging latrines, and others, were grouped as unimproved toilet facilities. The third group consisted of those who used an open field for defection and had neither an improved nor an unimproved form of toilet.

Wealth index of the household was computed using the principal component analysis on the basis of household assets (the number and kinds of consumer goods they own, ranging from a television to a bicycle or car, and housing characteristics such as source of drinking water, toilet facilities, and flooring materials). The computation was done separately for urban and rural resident as the household assets vary by residence. Household scores derived using principal components analysis were assigned for each usual household member. Finally, each person in the surveyed household were ranked by her or his score, and then divided into five equal categories, each comprising 20\% of the population. These categories were named as poorest, poorer, medium, richer, and richest. These categorizations and indices are created by the DHS.

### Data processing and analysis

A total of 1,068 children aged 12 to 23 months were retained after excluding those outside the specified age range from the EMDHS 2019 children dataset. Of these, 60 children had missing values for DPT1 vaccination status, leaving 1,008 children for the final analysis. Relevant variables were subsequently retrieved from this survey dataset for further analysis.

Because of the nature of the sampling design, all analyses were performed utilizing the complex sampling design adjustment approach and non-response rate. Accordingly, weighted prevalence of zero-dose immunization was computed and further combined with the GPS coordinates in each of the EMDHS 2019 clusters. As a result, the cluster level prevalence of zero-dose immunization was exported into ArcGIS to depict hot and cold spots of clusters.

Geographic variation in the prevalence of zero-dose immunization among EMDHS EAs was identified using spatial analysis, particularly auto-correlation and inverse distance weighted interpolation [24, 25]. Local Moran’s I statistic [24] as part of spatial auto-correlation was used to identify spatial patterns, hot spots and cold spots of zero-dose prevalence. Maps depicting spatial distribution and variations through out the country were constructed. In addition, as a complement to Moran’s I statistic, inverse distance weighted interpolation [25] was employed to estimate these distributions, facilitating the visualization of spatial patterns and enhancing the interpretability of spatial auto-correlation by generating a continuous surface that bridges statistical findings with practical spatial insights.

The standard binary logistic regression estimates are inadequate since the data originates from a complex survey design with stratification, clustering, and unequal weighting [26]. If the sampling design is not included in the analysis, the standard errors are likely be underestimated, perhaps leading to statistically significant findings when they are not [26–28]. As a result, the survey binary logistic regression model [26] was used to analyze data in order to account for the complex sampling design. The bivariable and multivariable survey binary logistic regression models were used to evaluate the associated factors of the zero-dose immunization. Bivariable analysis was used to examine the relationship between socio-demographic characteristics and the outcome variable. The multivariable analysis included all variables with *p*-value less than or equal to 0.25 in the bi-variable analysis. Variables with a *p*-value of less than 0.05 were considered statistically significant in multivariable analysis. Variances were computed using Taylor series linearization [29] to estimate the variance considering the sampling weight.

To demonstrate the strength of the association, the adjusted odds ratio (AOR) with the accompanying 95% confidence intervals were provided. These demonstrate whether each category of a variable raises (AOR > 1) or reduces (AOR < 1) the likelihood of having a zero-dose immunization relative to the reference category, after adjusting for all other independent factors.

ArcGIS Desktop version 8.0 was utilized for spatial analysis, while R was used for the remaining analyses. Specifically, the survey package of R [30] was used for the weighed analysis.

## Results

### Characteristics of the participants

A total of 1008 children aged 12 to 23 months old were retained for the final analysis after excluding missing observations for the outcome. The median age of the mothers/care givers was 27 years old with interquartile range of 23 to 32 (Table 1). The mean age of the children was about 17 months old with SD of 3.4.

**Table 1:** Weighted summary measures of the study participants in Ethiopia, 2019

Variable	Summary Measure
Mother’s/caregiver’s age (Years)	Median: 27 (IQR: 23–32)
Household size	Median: 5 (IQR: 4–7)
Round trip time (Minute) to water source	Median: 20.0 (IQR: 5.0–41.1)
Age of the child	Mean: 17.1 (SD = 3.4)
Timing of the first *ANC* visit	Median: 4 (IQR: 3–5)
Number of *ANC* visits	Median: 3 (IQR: 1–4)

### Magnitude of zero-dose immunization

The weighted prevalence of zero-dose immunization was computed long with the corresponding 95% confidence intervals at the national level and stratified by the factors (Table 2). Accordingly, 23.7% [95% CI: 18.7–28.5] of children aged 12–23 months in Ethiopia were zero-dose. The percentage of zero-dose children increases as the household size increases, that is 19.0% among children from a household of size 2–4 members and 31.6% among children from a household of size eight or more members. Similarly, there was observed difference in percentage of zero-dose immunization by place of residence, 29.7% in rural and 10.0% in urban areas. Furthermore, the percentage of zero-dose among children aged 12–23 months in Ethiopia decreases as the educational status of the mother/caregiver increases with highest percentage (33.5%) observed among those who don’t attended any formal education and the lowest percentage (4.9%) among those who attended secondary school and above. There was observed regional discrepancy in percentage of zero-dose children with the highest observed in Somali region (56.0%) and the smallest observed in Addis Ababa (3.7%).

**Table 2:** Weighted prevalence of zero-dose immunization by selected characteristics of the study participants in Ethiopia, 2019

Variable	Categories	% (95% CI)
Toilet facility	Improved	7.1 [0.0, 14.1]
	Un Improved	21.2 [15.9, 26.6]
	Open Field	32.6 [24.2, 41.0]
Mother’s/Caregiver’s Age	15–24	25.4 [16.6, 34.2]
	25–29	23.0 [15.5, 30.4]
	30–34	25.8 [17.2, 34.4]
	35–49	20.7 [12.3, 29.2]
Toilet Shared	Yes	16.2 [7.0, 25.4]
	No	20.7 [15.3, 26.1]
Household size	2–4	19.0 [12.8, 25.1]
	5–7	24.2 [17.6, 30.7]
	8+	31.6 [22.9, 40.3]
Region	Tigray	4.6 [0.1, 9.1]
	Afar	53.5 [40.7, 66.4]
	Amhara	15.6 [7.2, 24.0]
	Oromia	26.7 [16.6, 36.5]
	Somali	56.0 [41.8, 70.2]
	Benishangul Gumuz	10.7 [3.7, 17.7]
	SNNP	27.4 [18.8, 35.9]
	Gambela	23.7 [8.8, 38.5]
	Harari	36.8 [22.3, 51.3]
	Addis Ababa	3.7 [0.0, 8.5]
	Dire Dawa	4.8 [0.5, 9.1]
Residence	Urban	10.0 [3.3, 16.8]
	Rural	29.7 [23.4, 35.9]
Mother’s/Caregiver’s Education	Secondary/Higher	4.9 [0.4, 9.4]
	Primary	19.4 [12.2, 26.5]
	No Formal Education	33.5 [27.5, 39.6]
Type of water source	Improved	20.3 [15.1, 25.6]
	Un-Improved	30.7 [22.0, 39.5]
Time to water source (in minutes)	On Premise	4.2 [1.1, 7.3]
	0–30	26.8 [19.9, 33.7]
	31–60	27.3 [18.3, 36.4]
	>=61	36.1 [24.1, 48.0]
Mother’s/Caregiver’s Religion	Orthodox	14.5 [8.5, 20.5]
	Catholic	62.4 [35.9, 89.0]
	Protestant	25.1 [16.4, 33.8]
	Muslim	31.2 [21.3, 41.2]
	Traditional	35.4 [10.5, 60.4]
	Other	48.3 [43.5, 53.1]
HH Wealth index	Poorest	41.2 [29.8, 52.5]
	Poorer	24.2 [15.2, 33.2]
	Medium	30.3 [19.3, 41.3]
	Richer	22.0 [11.6, 32.4]
	Richest	5.2 [0.8, 9.7]
Mother’s/Caregiver’s Marital status	Currently in union	23.4 [18.4, 28.3]
	Never/formerly in union	30.6 [9.9, 51.2]
Child sex	Male	21.0 [15.3, 26.8]
	Female	26.2 [19.9, 32.4]
Institutional Delivery	Yes	8.9 [5.5, 12.3]
	No	41.2 [34.0, 48.5]
ANC1	Yes	14.9 [10.7, 19.1]
	No	50.4 [40.6, 60.2]
ANC4	Yes	8.8 [4.5, 13.0]
	No	35.6 [29.1, 42.2]
ANC8	Yes	3.3 [0.0, 9.2]
	No	24.2 [19.2, 29.2]
Total		23.7 [18.7–28.5]

### Spatial analysis of zero-dose immunization

**Fig. 1:**
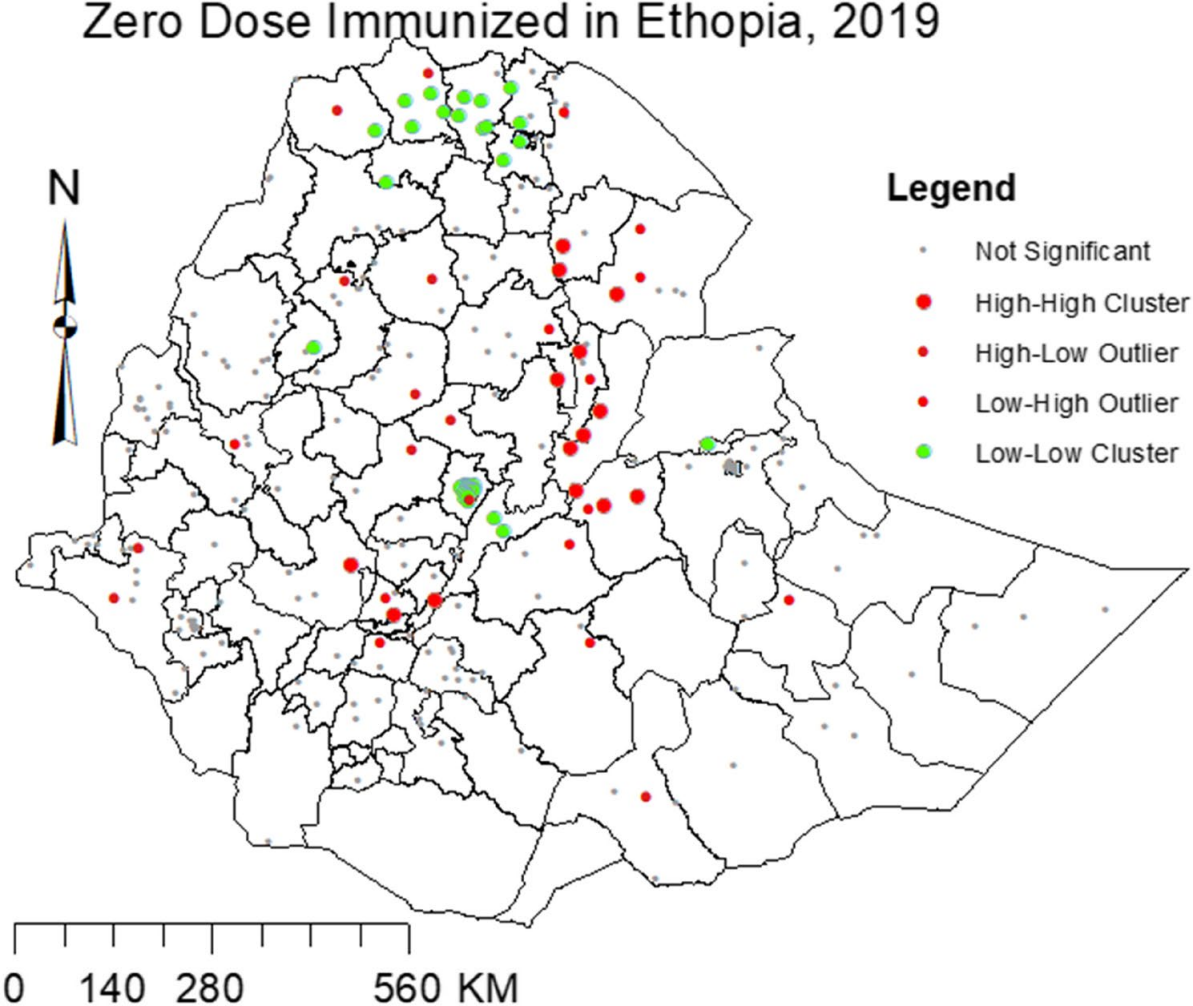
Cluster and hotspot of prevalence of zero-dose immunization in Ethiopia, 2019

The analytic output of Anselin Local Moran’s I divided into four primary groups: two for clusters and two for outliers. High-High (HH) and Low-Low (LL) clusters highlight similar enumeration regions with high and low prevalence respectively. High-Low (HL) and Low-High (LH) identifies outliers with high prevalence surrounded by EAs of low prevalence and vice versa. Figure 1 depicts a cluster of zero-dose immunization high values (hot spots) and low values (cold spots). This plot revealed the occurrence of hot spots of high prevalence of zero-dose immunization in southwestern and northeastern parts of Ethiopia.

Significant clusters of hot spots (high prevalence) and cold spots (low prevalence) of zero-dose immunization were identified. There were identified hot spots of high prevalence of zero-dose immunization in Ethiopia. This shows that in addition to the overall high prevalence of zero-dose immunization in the country, there was spatial variation. Accordingly, the hot spots of zero-dose immunization were observed in south-west (Jimma zone, Halaba zone and Kembata Tembaro zone) and northeast (West Hararge zone, Gabi zone/Zone3, North Shewa (AM) zone, Hari zone/Zone 5, Awsi zone/Zone 1, and Fanti zone/Zone 4) part of the country whereas cold spots of zero-dose immunization were observed in the central and northern parts of Ethiopia. Although no significant clusters of areas with high prevalence of zero-dose children were observed in Addis Ababa, a few outliers with high prevalence, surrounded by low-prevalence areas, were identified within the urban setting of Addis Ababa. Hence, further detailed investigations on spatial attributes leading to different effects on zero-dose immunization could be conducted.

**Fig. 2:**
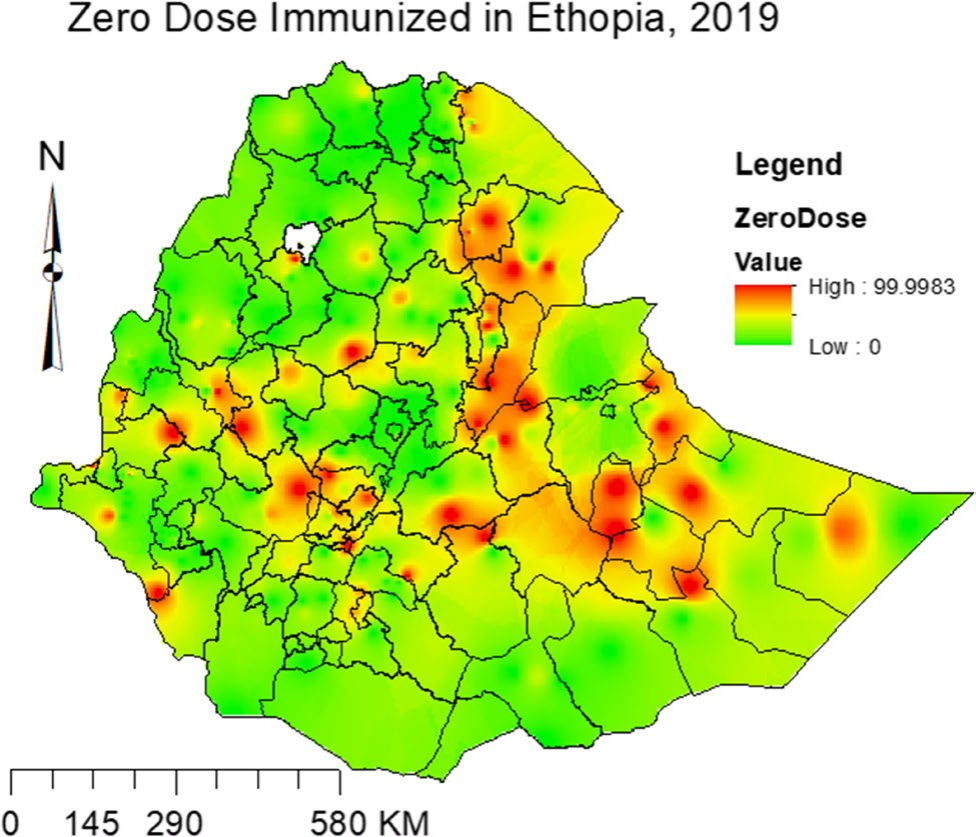
Predicted prevalence of zero-dose immunization in Ethiopia, 2019

In addition, the predicted prevalence of zero-dose immunization (Fig. 2) complemented the spatial pattern analysis by aligning predicted prevalence with observed clusters, where areas with high zero-dose prevalence exhibited similarly high predicted values and vice versa. This consistency reinforced the spatial patterns identified and provided a visual enhancement to the statistical findings. This alignment highlights clear identification of regions in targeting specific areas for intervention, making the findings more actionable for public health strategies.

### Associated factors of zero-dose immunization

Toilet facility, mother age, toilet shared, and marital status were removed as they showed *p*-value > 0.25 from the bivariable binary survey regression analysis. Additionally, variables atleast one ANC visit, atleast eight ANC visit, time to water source were excluded accounting multicollinearity from the theoretical point of view.

From the final multivariable analysis, the variables region, residence, sex of the child, four or more ANC visits and institutional delivery were found to be statistically significant associated factors of zero-dose immunization among children in Ethiopia (Table 3). Rural children were 2.55 [95%: 1.05, 6.22] times likely to be zero-dose as compared to their counter urban residents. Female children were nearly two times 1.78 (95% CI: 1.09, 2.91) likely to be zero-dose as compared to male children. Those children who were not born at the health institution were 4.12 (95% CI: 2.39, 7.08)- and those whose mother had not completed four or more ANC visits were 2.55 (95% CI: 1.37, 4.75)- times likely to be zero-dose among children aged 12–23 months in Ethiopia.

**Table 3:** Associated factors of zero-dose immunization among children 12–23 months in Ethiopia, 2019

Variable	Categories	COR (95% CI)	AOR (95% CI)
Household size	2–4 (ref.)		
	5–7	1.36[0.84, 2.21]	1.03[0.52, 2.03]
	8+	1.98[1.18, 3.30]	0.96[0.51, 1.80]
Region	Tigray(ref.)		
	Afar	24.04[7.56,76.44]	26.63[4.89,145.17]
	Amhara	3.86[1.15,13.03]	3.72[0.88, 15.72]
	Oromia	7.55[2.38,23.92]	6.72[1.37, 33.06]
	Somali	26.55[8.13,86.74]	17.63[2.89,107.68]
	Benishangul Gumuz	2.50[0.71,8.89]	3.78[0.69, 20.78]
	SNNP	7.86[2.56,24.07]	8.13[1.70, 38.95]
	Gambela	6.47[1.73,24.25]	13.71[2.15, 87.30]
	Harari	12.15[3.63,40.62]	18.42[3.05,111.27]
	Addis Ababa	0.81[0.15,4.35]	8.21[0.85, 79.36]
	Dire Dawa	1.05[0.26,4.24]	1.18[0.15, 9.24]
Residence	Urban (ref.)		
	Rural	3.79[1.69,8.51]	2.55[1.05,6.22]
Mother’s Education	Secondary/Higher (ref.)		
	Primary	4.67[1.71,12.78]	1.57[0.51, 4.84]
	No Formal Education	9.83[3.63,26.60]	1.92[0.62,5.97]
HH Wealth index	Poorest (ref.)		
	Poorer	0.46[0.23,0.92]	0.84[0.34, 2.06]
	Medium	0.62[0.32,1.21]	1.28[0.55, 2.99]
	Richer	0.40[0.19,0.84]	1.30[0.46,3.70]
	Richest	0.08[0.03,0.22]	0.79[0.18,3.48]
Child sex	Male (ref.)		
	Female	1.33[0.90,1.97]	1.78[1.09, 2.91]
Type of water source	Improved	0.58[0.35,0.94]	0.96[0.56, 1.64]
	Un-Improved (ref.)		
Religion	Orthodox (ref.)		
	Catholic	9.83[2.86,33.72]	1.17[0.22,6.37]
	Protestant	1.98[1.04,3.77]	1.00[0.38,2.59]
	Muslim	2.69[1.37,5.27]	1.15[0.45,2.92]
	Traditional	3.24[0.97,10.86]	1.22[0.35,4.26]
	Other	5.53[3.28,9.33]	0.70[0.22,2.19]
Institutional Delivery	Yes (ref.)		
	No	7.18[4.70,10.98]	4.12[2.39,7.08]}
ANC4	Yes (ref.)		
	No	5.76[3.35,9.9]	2.55[1.37,4.75]

## Discussion

This study identified the prevalence, spatial distribution, and associated factors of zero-dose immunization among children in Ethiopia.

The overall weighted prevalence of zero-dose immunization at national level in Ethiopia was 23.7% [95% CI: 18.7–28.5]. The figure is in agreement with another study conducted in Togo that reported the prevalence of “zero-dose” in children aged 12–23 months was 26.9% (95% CI = 23.50-30.55) [31]. However, our finding is lower than findings of other studies in Ethiopia [15, 16], the discrepancy could be for the difference in consideration of vulnerable groups and wider age group for their study. However, the findings on an overall zero-dose immunization prevalence of 26.8% looks masking significant spatial disparities across Ethiopia, the prevalence ranges from just 3.7% in Addis Ababa to alarmingly high levels of 53.5% in Afar and 56% in Somali regions. These regional and local variations, were supported by spatial mapping of hot spots, underscore critical gaps in zero-dose.

From the spatial analysis, a significant geographic variation was detected. Accordingly, geographically tailored intervention to explore more and take action in the identified hot spot areas of zero-dose immunization including southwest (Jimma zone, Halaba zone and Kembata Tembaro zone) and northeast (West Hararge zone, Gabi zone/Zone3, North Shewa (AM) zone, Hari zone/Zone 5, Awsi zone/Zone 1, and Fanti zone/Zone 4), parts of Ethiopia. In addition, the predicted prevalence of zero-dose immunization complemented the spatial pattern analysis, this consistency reinforced the spatial patterns identified and provided a visual enhancement to the statistical findings. This alignment highlights clear identification of regions in targeting specific areas for intervention, making the findings more actionable for public health strategies. In contrast, further investigation in the cold spots area might help to take a lesson on how zero-dose immunization is low. Notably, outliers with a high prevalence of zero-dose children were also observed in urban areas like Addis Ababa, highlighting the existence of localized inequities even within better-performing regions. Furthermore, future studies applying more advanced prevalence estimation methods using more survey data and advanced spatial analysis could help to get more reliable results. The observed spatial pattern could also be further stratified by the identified factors to identify the vulnerable individuals.

In addition to the spatial/geographic variation in the occurrence of zero-dose immunization, being rural resident, being female children, children not delivered at the health institution, and children from a mother did not complete four or more ANC visits were more likely to be zero-dose as compared to their counter parts where these could be stratify children in the hotspot or cold spot areas to reach out more vulnerable ones.

Accordingly, in agreement with numerous studies this study showed a child being rural resident were more likely to be zero-dose. In contrast, some findings showed urban/rural living was not a significant driver of zero-dose status in the global context [21] whereas in LMICs, living in rural area was associated with a lower likelihood of being zero-dose. The variation in zero-dose immunization among urban and rural residents might be because of the supply side factors and the demand side factors. Low awareness and perception about the zero-dose immunization in rural areas from the demand side and long-distance travel times, and poor infrastructure in the rural areas among the demand side factors might be the cause [32]. Thus, priority should be given for children in the rural areas as compared to urban children in Ethiopia.

After adjusting for other factors, child’s sex was significantly associated with zero-dose immunization. However, in the global level numerous studies showed that there is no significant difference in immunization coverage for boys and girls [21]. The results from studies conducted in Ethiopia [33, 34] showed the difference between male and female might be from cultural differences in the community. Sex related barriers might also have an indirect impact on zero-dose immunization. Social and cultural values and norms towards sex difference in many societies, can raise difference in the chances of zero-dose among children by sex. A study in India [35] suggested addressing deep seated social and cultural issues responsible to minimize the extent of zero-dose immunization. Further, this study also suggested campaigns to raise awareness on cultural and social barriers with improvement in vaccine delivery strategies with a focus on eliminating zero-doze immunization status. Continuing research is needed in order to identify social and cultural values and norms, myths, misconceptions and fears so as to design effective policies reducing inequities in zero-dose immunization and other health practices among boys and girls. The research could also be primarily in the identified hot spot areas of zero-dose immunization in this study, in Ethiopia. Thus, it is vital that immunization programs consider these barriers and address the people and societies in their communities.

Home delivery was identified as a factor associated with an increased likelihood of zero-dose immunization status. A similar study showed that children delivered at home were more likely to be zero-dose compared with children delivered at health facilities [21, 36]. This conclusion may be explained by the fact that a mother giving birth at home misses the vaccinations administered to the child at birth, as well as any counseling or scheduling related to vaccinations. This is in line with earlier studies, which were more interested in cases of both complete and incomplete vaccinations than in zero-dose cases. A context-specific strategy, including establishing micro-health centers or working with traditional birth attendants to identify, track, and vaccinate infants from birth, will greatly increase immunization coverage and the battle against diseases that can be prevented by vaccination in under served communities.

Further, in this study children from a mother did not complete four or more ANC visits were more likely to be zero-dose as compared to their counter parts. A similar finding was reported, children of mothers who did not receive any ANC visits were nearly 2.5 times likely to be zero-dose than children of mothers who received at least four ANC visits [21]. Further, a review study [32] showed that maternal care (ante-natal) factors were identified as the risk factors for the zero-dose immunization status. Receiving antenatal care was associated with higher vaccination rates. The ANC utilization might be because of lack of access or non-utilization, so both aspects should be considered to improve level of zero-dose immunization status in Ethiopia.

### Limitations

There are a few limitations to this study. First, while we identified factors associated with zero-dose immunization, there may be unmeasured explanatory variables not captured in the DHS dataset, such as cultural beliefs, vaccine supply issues, and community-level factors. Second, the survey data utilized was six years old, which may not fully reflect the current immunization status, even though it was the most recent national data available. Third, while spatial analysis highlighted areas of high zero-dose, the inability to apply small area estimation (SAE) techniques due to the unavailability of recent auxiliary data, limited the granularity of our estimates. Although SAE techniques [37] can enhance precision by leveraging multi-source data, their implementation demands significant computational resources and comprehensive auxiliary variables, which were not available. This trade-off highlights the challenge of balancing methodological sophistication with practical feasibility when aiming to achieve reliable and precise estimates under data and resource constraints. Therefore, readers are encouraged to interpret the findings in light of these limitations.

## Conclusion

There is high prevalence of zero-dose immunization among 12–23 months old children in Ethiopia, with notable spatial disparities across the country. Hot spots of zero-dose children were identified in the southwestern and northeastern parts, while some urban areas, including parts of Addis Ababa, showed an unexpectedly high zero-dose prevalence, suggesting localized inequalities. Key factors associated with zero-dose status included rural residence, female sex, birth outside health facilities, and mothers not receiving four or more antenatal care visits. These findings underscore the importance of geographically and demographically targeted public health interventions. Future studies could enhance precision by incorporating advanced spatial modeling and small area estimation approaches, provided that relevant auxiliary data are available.

## Data Availability

Minimal data that support the findings of this study can be accessed from the correspondence upon reasonable request. The data are not publicly available due to privacy or ethical restrictions.

## Abbreviations

ANC: Antenatal Care
ANC1: Atleast one ANC visit
ANC4: Atleast four ANC visits
ANC8: Atleast eight ANC visits
AOR: Adjusted Odds Ratio
CI: Confidence Interval
COR: Crude Odd Ratio
CSA: Central Statistics Agency
COVID: Corona Virus Disease
DHS: Demographic and Health Survey
DTP: Diphtheria, Tetanus, and Pertusis
DTP1: First dose of the Diphtheria, Tetanus, and Pertusis
EDHS: Ethiopia DHS
EMDHS: Ethiopian Mini DHS
EAs: Enumeration Areas
EPHI: Ethiopian Public Health Institute
FMoH: Federal Ministry of Health
GPS: Global Positioning System
IQR: Inter-Quartile Range
LMICs: Lower and Middle Income Countries
PNC: Postanatal Care
SD: Standard Deviation
SNNP: Southern Nations, Nationalities and People
VIP: Ventilated Improved Pit
VPD: Vaccine Preventable Diseases
WHO: World Health Organization
